# Supplementary figures and images for: Evaluating the Mode of Antifungal Action of Heat-Stable Antifungal Factor (HSAF) in Neurospora crassa
Source: J Fungi (Basel). 2022 Mar 1;8(3):252. doi: 10.3390/jof8030252 (PMC8951606; doi:10.3390/jof8030252)

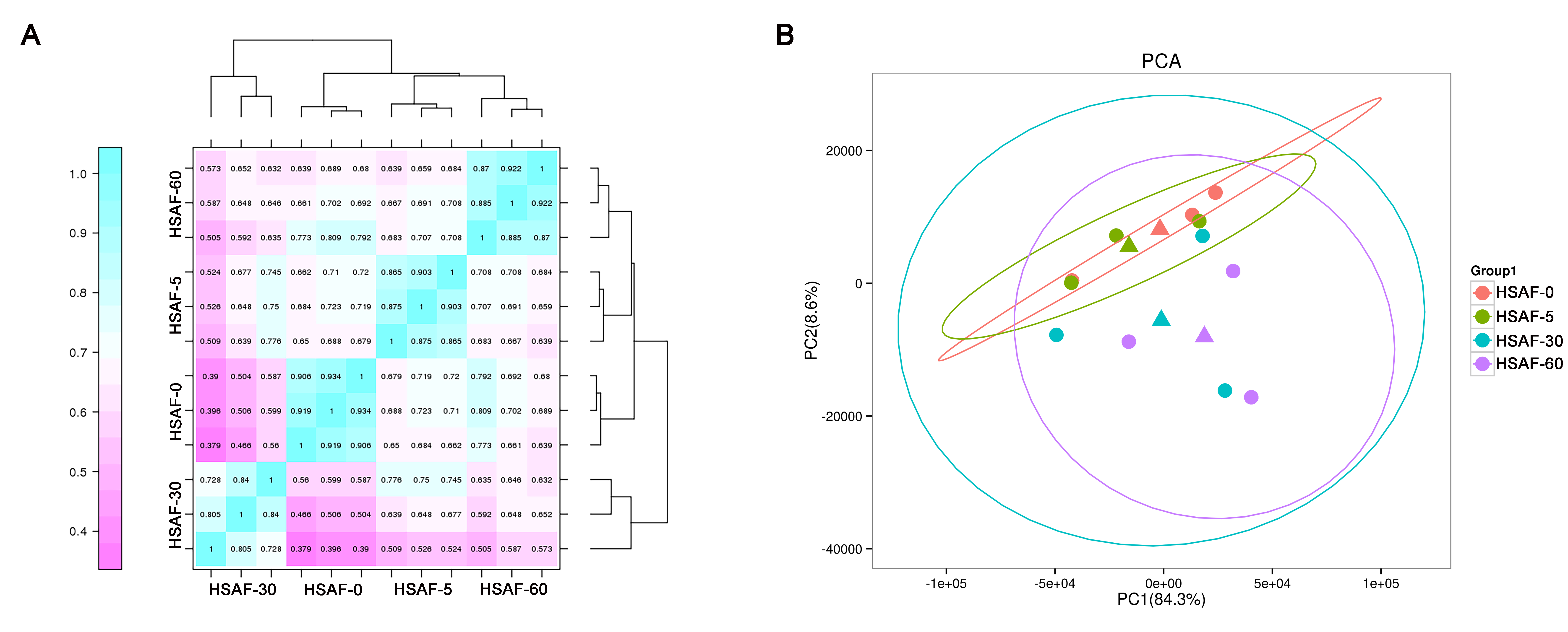

Supplement: Supplementary file 1 [file jof-08-00252-s001.zip › Figure S1.tif]

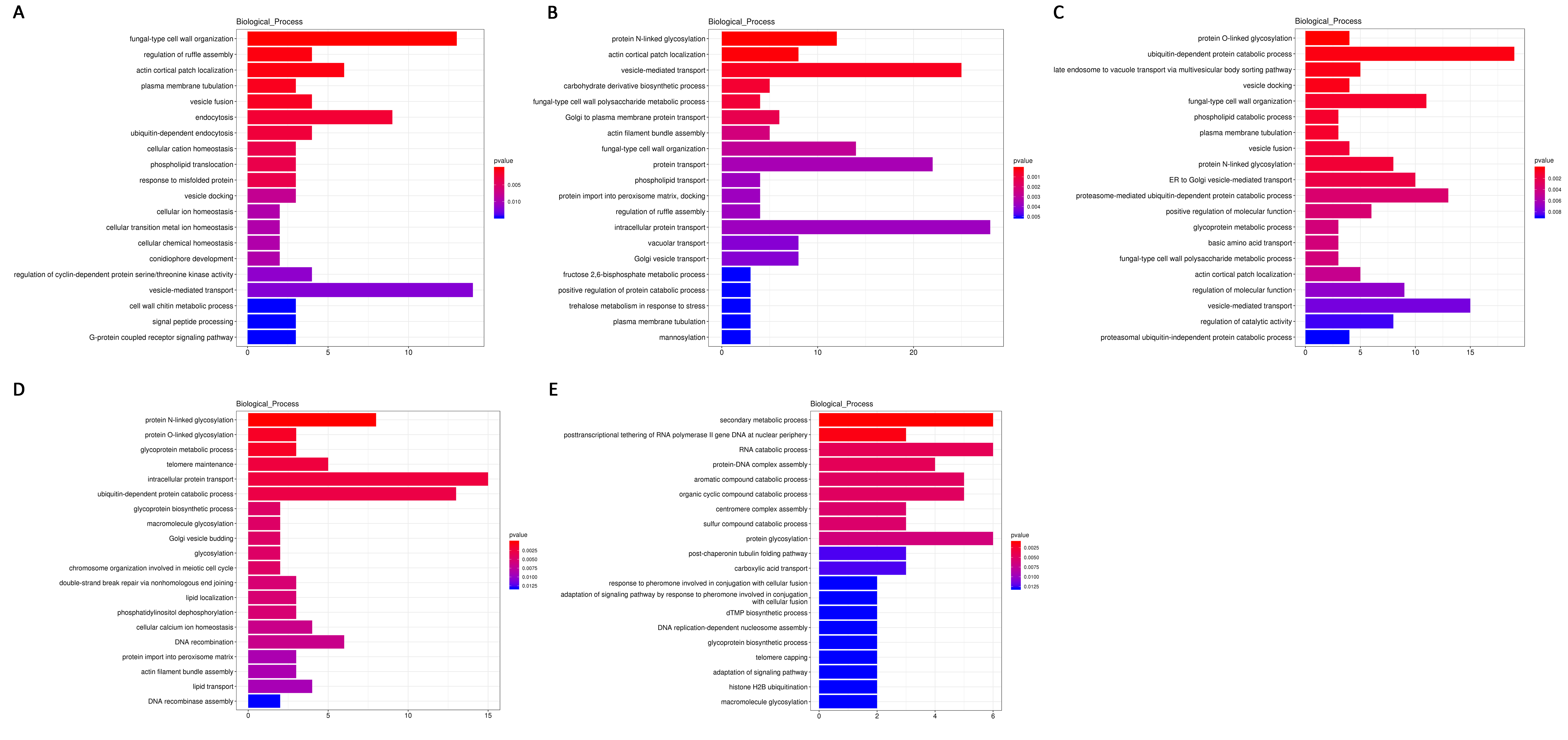

Supplement: Supplementary file 1 [file jof-08-00252-s001.zip › Figure S2.tif]

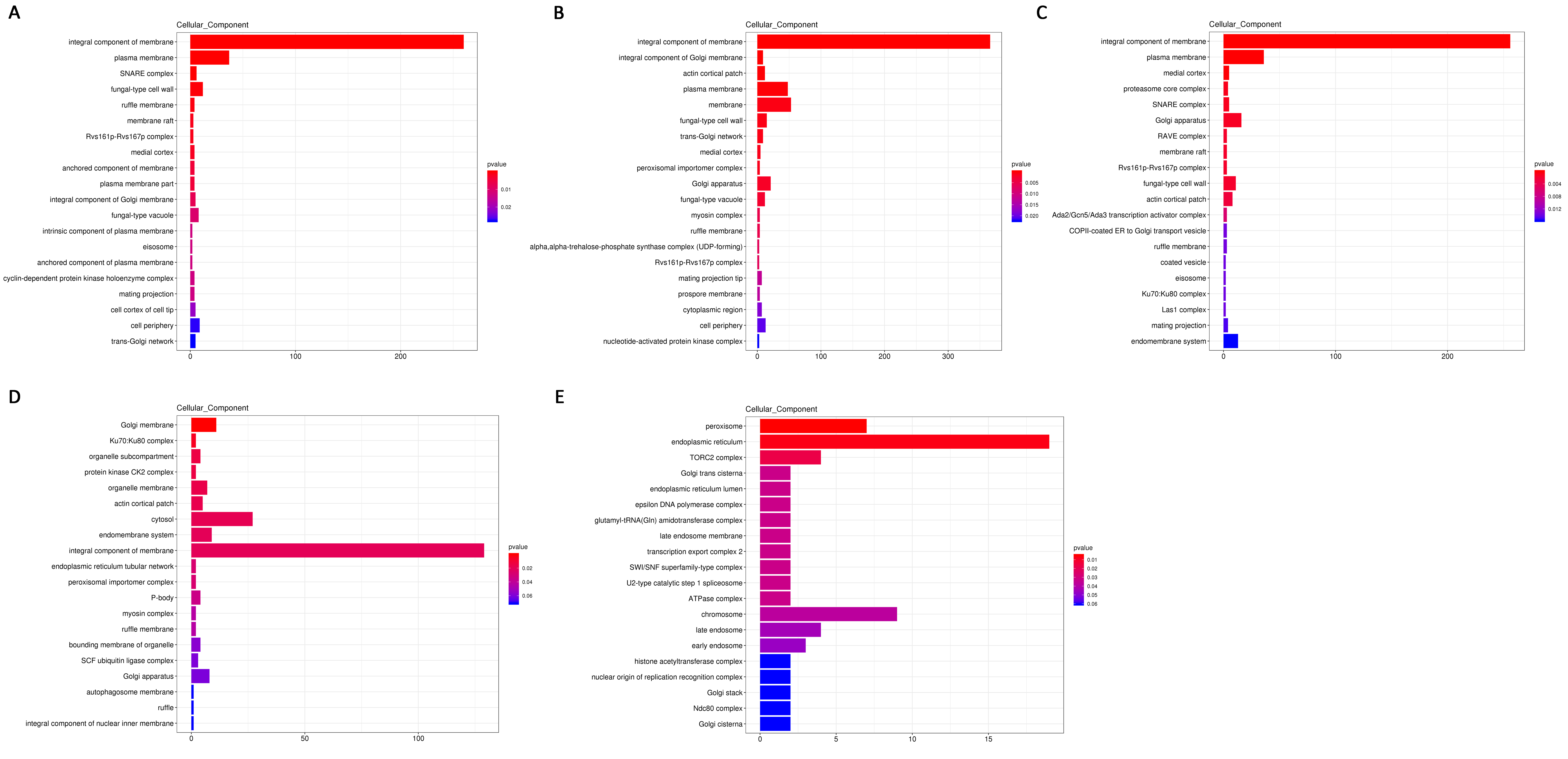

Supplement: Supplementary file 1 [file jof-08-00252-s001.zip › Figure S3.tif]

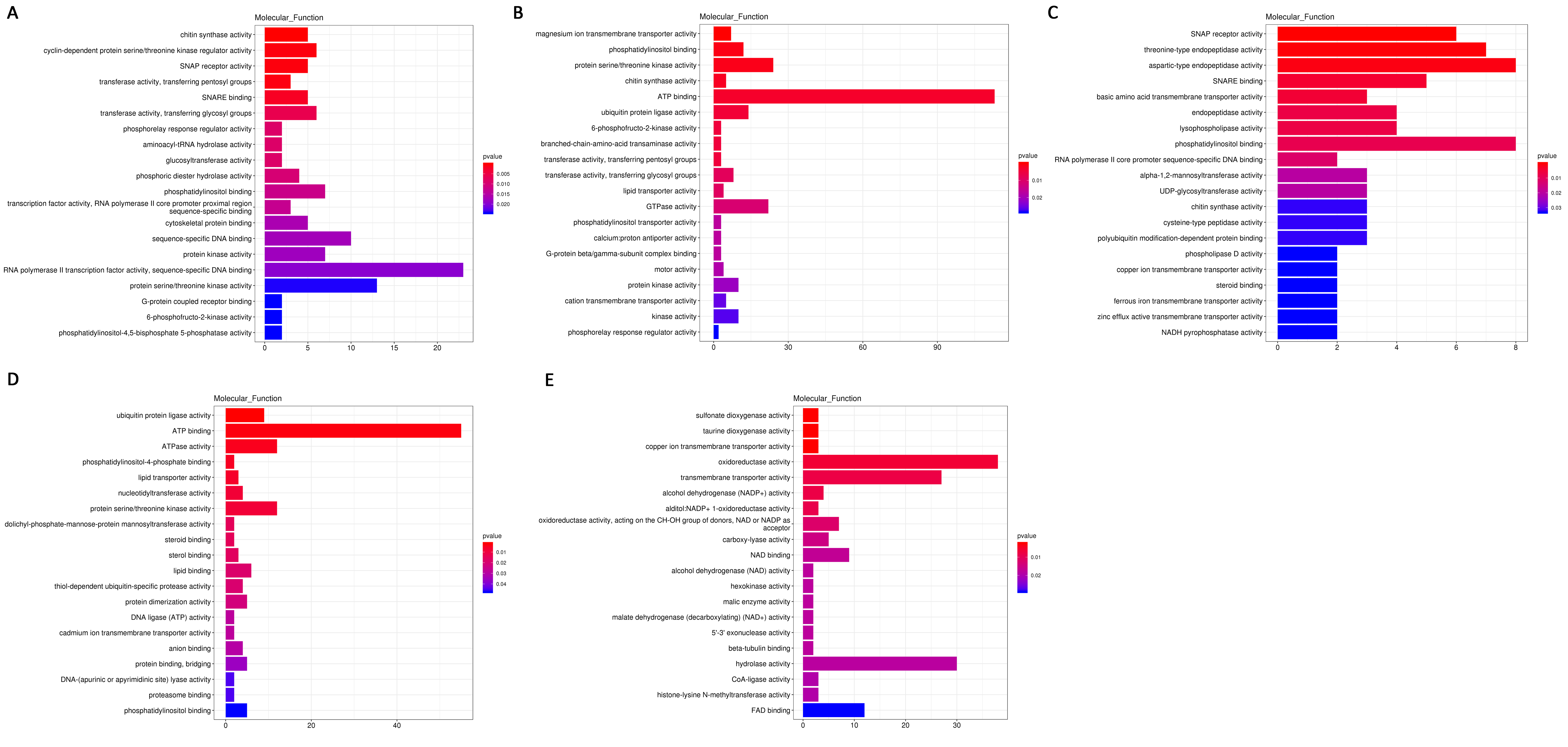

Supplement: Supplementary file 1 [file jof-08-00252-s001.zip › Figure S4.tif]

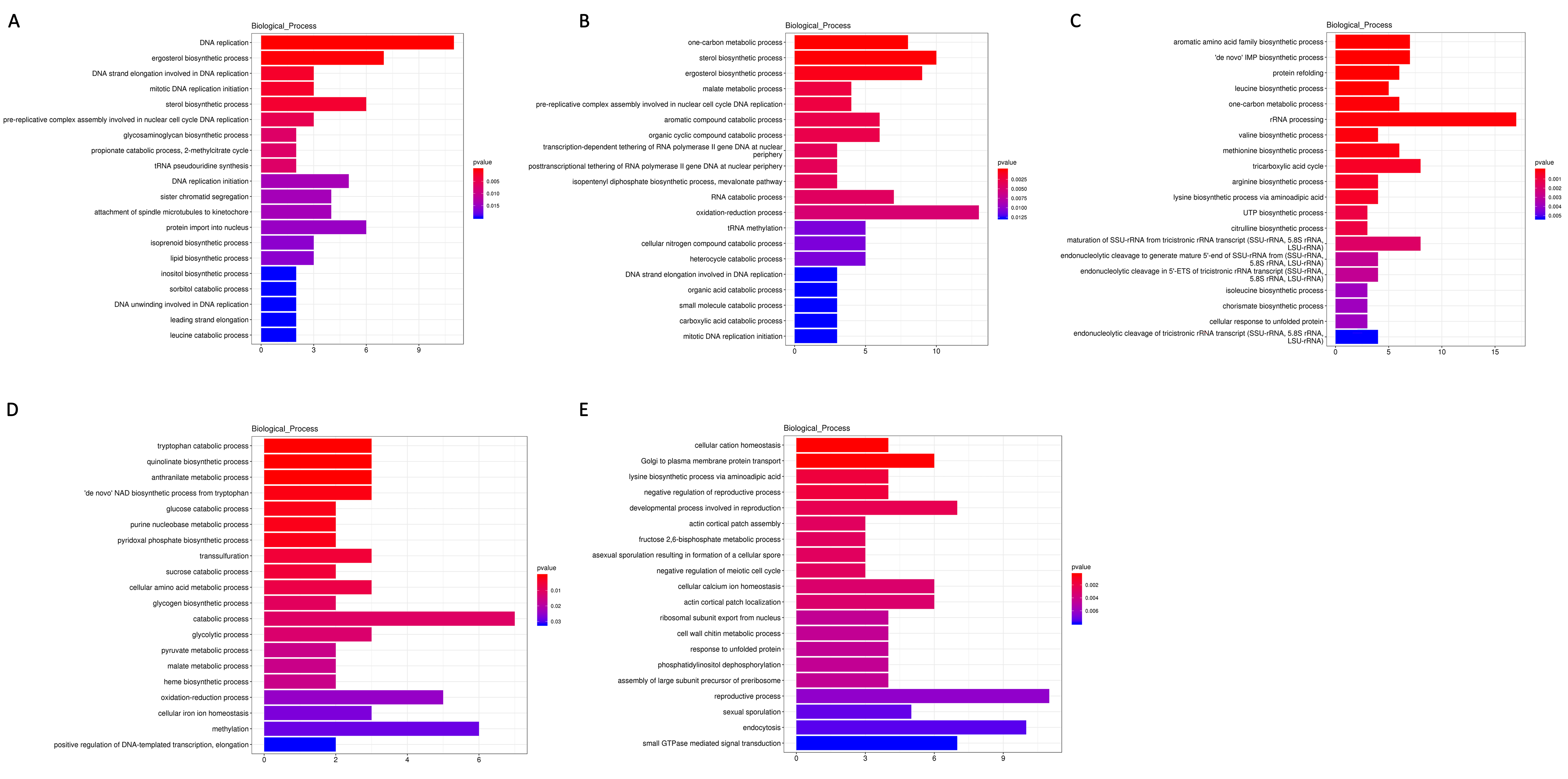

Supplement: Supplementary file 1 [file jof-08-00252-s001.zip › Figure S5.tif]

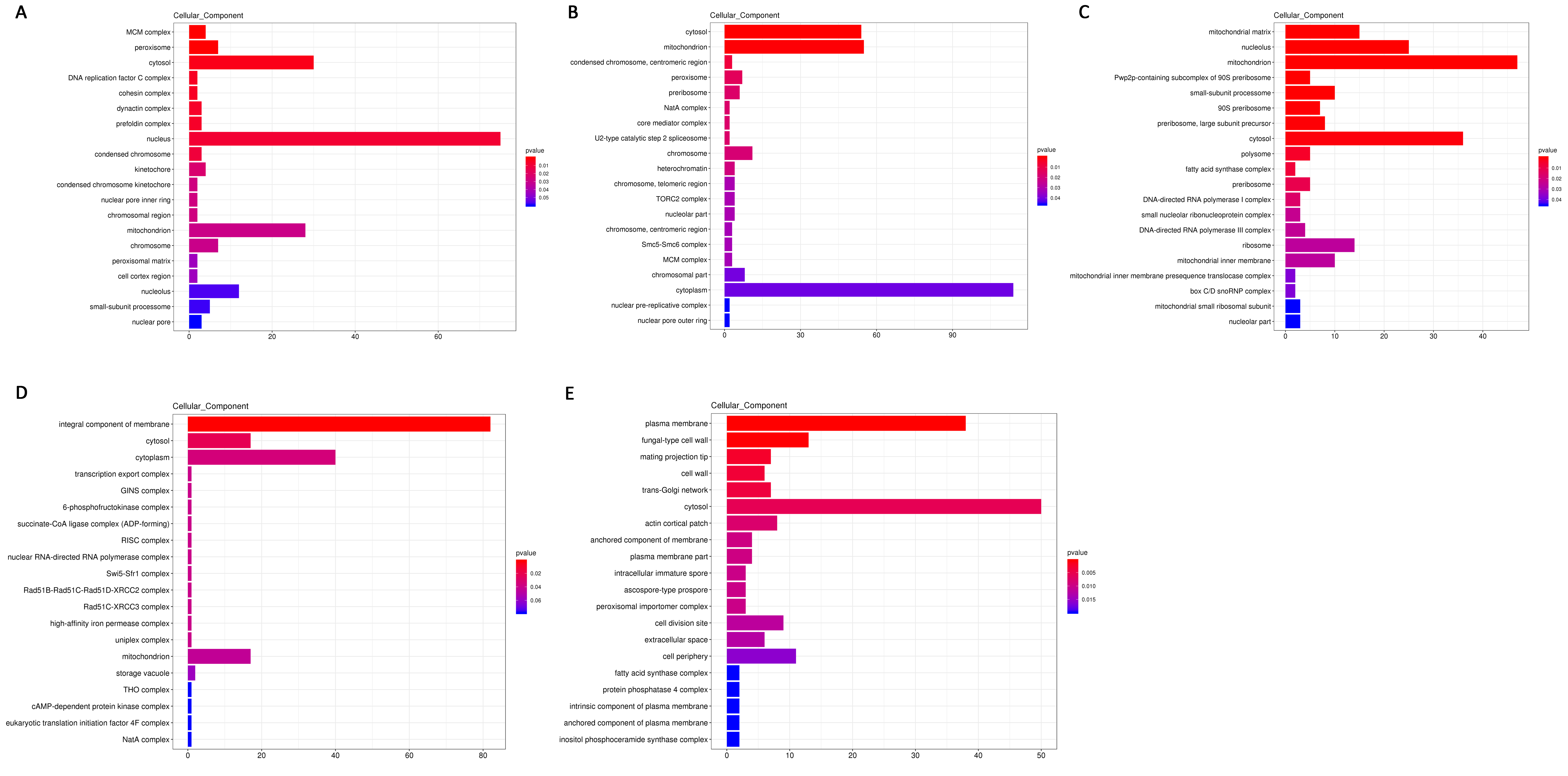

Supplement: Supplementary file 1 [file jof-08-00252-s001.zip › Figure S6.tif]

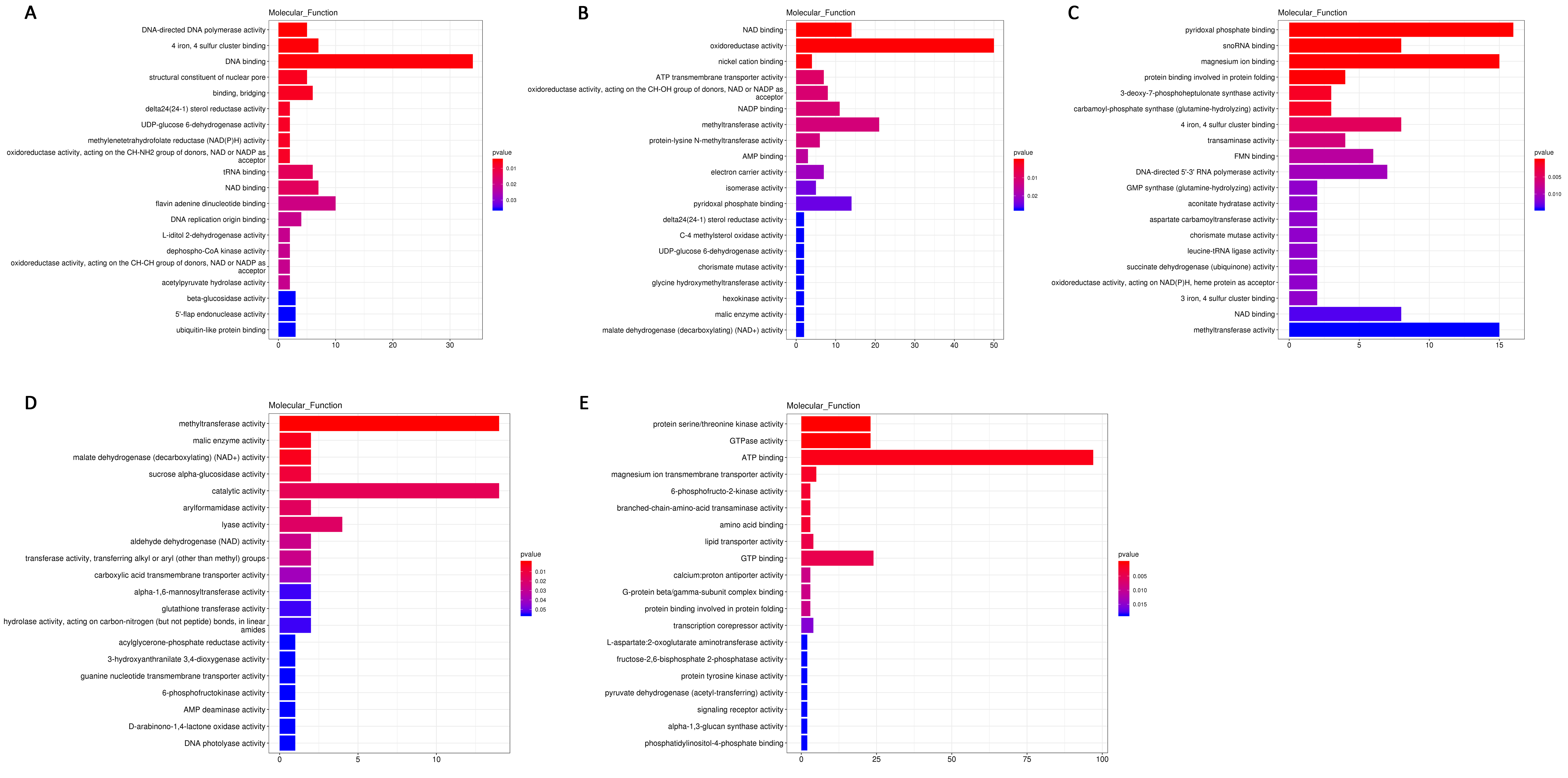

Supplement: Supplementary file 1 [file jof-08-00252-s001.zip › Figure S7.tif]
